# Rapid Detection of Pathogens in Wound Exudate via Nucleic Acid Lateral Flow Immunoassay

**DOI:** 10.3390/bios11030074

**Published:** 2021-03-06

**Authors:** Anna Brunauer, René D. Verboket, Daniel M. Kainz, Felix von Stetten, Susanna M. Früh

**Affiliations:** 1 Laboratory for MEMS Applications, IMTEK—Department of Microsystems Engineering, University of Freiburg, Georges-Koehler-Allee 103, 79110 Freiburg, Germany; anna.brunauer@imtek.uni-freiburg.de (A.B.); daniel.kainz@imtek.uni-freiburg.de (D.M.K.); felix.von.stetten@hahn-schickard.de (F.v.S.); 2 Department of Trauma-, Hand- and Reconstructive Surgery, University Hospital Frankfurt, Johann Wolfgang Goethe University, Theodor-Stern-Kai 7, 60590 Frankfurt am Main, Germany; Rene.Verboket@kgu.de; 3 Hahn-Schickard, Georges-Koehler-Allee 103, 79110 Freiburg, Germany

**Keywords:** recombinase polymerase amplification, nucleic acid lateral flow immunoassay, point-of-care diagnostics, paper-based detection, nucleic acid amplification test, wound exudate, wound infection

## Abstract

The rapid detection of pathogens in infected wounds can significantly improve the clinical outcome. Wound exudate, which can be collected in a non-invasive way, offers an attractive sample material for the detection of pathogens at the point-of-care (POC). Here, we report the development of a nucleic acid lateral flow immunoassay for direct detection of isothermally amplified DNA combined with fast sample preparation. The streamlined protocol was evaluated using human wound exudate spiked with the opportunistic pathogen *Pseudomonas aeruginosa* that cause severe health issues upon wound colonization. A detection limit of 2.1 × 10^5^ CFU per mL of wound fluid was achieved, and no cross-reaction with other pathogens was observed. Furthermore, we integrated an internal amplification control that excludes false negative results and, in combination with the flow control, ensures the validity of the test result. The paper-based approach with only three simple hands-on steps has a turn-around time of less than 30 min and covers the complete analytical process chain from sample to answer. This newly developed workflow for wound fluid diagnostics has tremendous potential for reliable pathogen POC testing and subsequent target-oriented therapy.

## 1. Introduction

Wound infections are a major problem for patients and the healthcare system. Surgical site infections (SSIs), for example, are responsible for about 24% of all health care associated infections in Germany. In the United States, the overall incidence of SSIs has been estimated to be about 2–5% (the rates can vary substantially depending on the procedure), corresponding to approximately 160,000–300,000 SSIs per year. SSIs lead to an increased length of hospitalization and higher morbidity rates, thereby posing a significant financial burden on public healthcare systems [1,2,3].

Thus, there is a clear need for methods that enable a fast and early detection of wound infection at the point-of-care (POC). Thus far, the gold standard for most infectious diseases is the tedious and time-consuming isolation and identification of pathogens in culture. Pathogen identification by nucleic acid amplification tests (NAATs) is faster and the method of choice for the detection of difficult-to-culture or non-cultivable microorganisms. NAATs like the polymerase chain reaction (PCR) are rather expensive, requiring specialized devices and sophisticated infrastructure [4]. A rapid test for pathogen detection and identification would provide a decision tool for a fast target-oriented therapy using specific antibiotics and antimicrobial substances, substantially advantageous compared to therapy decisions based on clinical presentation and empiric treatment only. This can improve the clinical outcome significantly and, in addition, prevent overuse of certain antibiotics, which can lead to resistance formation and other side-effects [5,6,7].

Wound exudate can be collected in a non-invasive way and is an attractive sample material for the detection of pathogens in infected wounds [8,9]. This bodily fluid is derived from plasma, and is comprised of a high amount of protein, inflammatory cells and other components. The high complexity and heterogeneity of this particular sample matrix may inhibit amplification reactions and makes the direct analysis of pathogens challenging [10]. Therefore, the amplification method of choice needs to be robust against known inhibitors, and the validity of the test result has to be tightly controlled by including an internal amplification control (IAC) [11].

The ideal POC test would allow the qualitative detection and identification of pathogens in wound exudate with a minimum of hands-on steps and a simple readout. Furthermore, the approach needs to cover the entire process chain consisting of: sample preparation (bacterial lysis), sample processing (target DNA amplification) and detection (of the amplified product) [12,13]. In recent years, several strategies have been developed for the rapid detection of pathogens in bodily fluids based on microfluidic [14,15,16] and biosensor approaches [17,18,19]. In this regard, lateral flow assays (LFAs) are highly attractive tools because they are inexpensive to produce and widely accepted by users and regulatory authorities [8,20,21]. The combination of paper-based microfluidic technology with isothermal nucleic acid amplification methods significantly contributed to the development of paper-based POC NAATs [22]. Initial approaches have been developed for the detection of pathogens from complex samples like whole blood [23,24,25,26], cerebrospinal fluid [27], saliva [28,29], swab samples [30,31,32,33], semen [34], or stool [35,36]. To the best of our knowledge, paper-based detection of pathogens in wound exudate has not been demonstrated so far.

Among the isothermal amplification methods that can be interacted with LFA-based analysis, especially the recombinase polymerase amplification (RPA) has gained in importance as a cost-effective and reliable technology for POC diagnostics [37]. In the last years several articles were published that successfully combined RPA and LFA [36,38,39,40,41,42,43,44,45,46,47,48,49,50,51,52,53,54,55,56,57,58,59]. However, only a minority of them showed direct amplification of target nucleic acids from crude lysate [45,46,47,48,49,50]. Most of these publications used purified nucleic acids [38,39,40,41,42,43,44] or commercial nucleic acid isolation kits [36,51,52,53,54,55,56,57,58,59] that require at least 6–13 hands-on steps and/or substantial sample preparation time (10–60 min), which adds to the total process time. Thus, these workflows do not meet the requirements of a rapid and simple POC test.

To address the need for a reliable POC test for the detection of pathogens in wound exudate, we developed a streamlined protocol with only three simple hands-on steps and a sample-to-result turnaround time of less than 30 min. The simplicity of the novel paper-based approach for wound fluid analysis is a result of integrating compatible reactions into a single reaction pathway. The assay strategy reported here combines lysis of the pathogens via bead beating, amplification of the genomic DNA (gDNA) from crude lysate via RPA and detection of the amplicon via nucleic acid lateral flow immunoassay (NALFIA). The workflow was tested and evaluated using human wound exudate spiked with *Pseudomonas aeruginosa* (*P. aeruginosa*).

*P. aeruginosa* is one of the most common pathogens that cause infection and delay healing in acute or chronic wounds [60,61,62]. In general, it is believed that a microbial load of >10^5^ colony forming units (CFU) per g of tissue, or mL of fluid is required for a wound infection and thus has been accepted by many as a critical value for diagnosing wound infection [63,64,65,66]. Therefore, a qualitative rapid test for the detection of pathogens in wound exudate with a limit of detection (LOD) of 10^5^ CFU per mL would offer a fast decision tool to (1) decide if a wound is infected and (2) start a target-oriented therapy using specific antibiotics. The here described approach serves to successfully detect *P. aeruginosa* in less than 30 min, achieving a LOD of 2.1 × 10^5^ CFU per mL in wound exudate.

## 2. Materials and Methods

### 2.1. Reagents

The recombinase polymerase amplification kit (TwistAmp^®^ nfo kit) was obtained from TwistDx Limited (Cambridge, UK). Primers and probes were obtained from Biomers (Ulm, Germany) and the internal amplification control DNA was purchased from BioCat GmbH (Heidelberg, Germany). The sheep anti-digoxigenin antibody was obtained from Bio-Rad AbD Serotec GmbH (Puchheim, Germany) and Polystreptavidin (Polystrept R) was obtained from BioTeZ Berlin Buch GmbH (Berlin, Germany). The rabbit anti-DNP antibody, donkey anti-sheep IgG antibody and the carboxylate-modified microspheres (FluoSpheres^TM^, 0.2 µm, red fluorescent (580/605)) were purchased from Life Technologies GmbH (Darmstadt, Germany). The lateral flow dipstick material (Backing card, nitrocellulose membrane CN140 and absorption pad CF5) was obtained from Kenosha (Amstelveen, The Netherlands), Sartorius AG (Goettingen, Germany), and GE Healthcare Life Science (Freiburg, Germany), respectively. Glass beads (0.1 mm) were purchased from Scientific Industries Inc. (Bohemia, NY, USA). 

### 2.2. Bacterial Strains and Culture Media

*P. aeruginosa* (DSM 1117) and its genomic DNA were obtained from DSMZ GmbH (Braunschweig, Germany). For specificity validation experiments, the following panel was used: *Staphylococcus aureus* (*S. aureus*, DSM 25923), *Staphylococcus epidermidis* (*S. epidermidis*, DSM 1798), *Streptococcus agalactiae* (*S. agalactiae*, DSM 2134), *Escherichia coli* (*E. coli*, DSM 6897), *Klebsiella pneumoniae* (*K. pneumoniae* DSM 30104), *Enterococcus faecalis* (*E. faecalis*, DSM 2570), and *Proteus mirabilis* (*P. mirabilis*, DSM 4479). All microorganisms were grown on LB agar plates and LB broth at 37 °C. Bacterial concentration was determined via serial dilution plating on LB agar plates. For *P. aeruginosa*, OD_600_ measurements were conducted, followed by plating of serial dilutions of the culture to correlate both parameters by linear regression analysis (see supplementary data Figure S1).

### 2.3. Spiked Wound Exudate

Wound exudate was taken from patients after informed consent and approval of the local ethics committee (Ethik-Kommission des Fachbereichs Medizin der Johann Wolfgang Goethe-Universität, Project number 111/17; Ethik-Kommission der Albert-Ludwigs-Universität Freiburg, Project number 28/17). Exclusion criteria were age younger than 18 years, urgent administration of major intravenous volume resuscitation, corticoid therapy, preoperative chemotherapy or radiotherapy, and infectious diseases. To keep the wound exudate comparable, only non-infected patients with a distal radial fracture and subsequent open reduction and internal fixation with wound drainage were included in the study. Sample collection was performed by puncturing the silicone tubes leading to the collection container. About 10 mL of fresh wound fluid was drawn off in a sterile syringe and placed directly on ice after the procedure. After collection, cellular content was removed by centrifuging at 800× *g* for 10 min in a refrigerated centrifuge. The wound exudate was then aliquoted into screw-cap tubes and stored at −80 °C until use. Prior to the measurements, the aliquots were gently thawed in a water bath. Due to the high heterogeneity of the biological sample wound exudate was pooled and spiked with *P. aeruginosa* (1.5 ± 0.4 × 10^4^–1.5 ± 0.4 × 10^7^ CFU/mL) where indicated. 

### 2.4. Procedure of Bacterial Lysis

Bead beating was used to lyse the bacterial cells. To this end, 800 µL cell suspension (bacteria in PBS or wound exudate containing 10% (*v/v*) LB broth) was transferred into 2-mL top screw tubes containing 800 mg glass beads (0.1 mm). Lysis was performed using the Precellys^®^ bead-beating homogenizer (Bertin Technologies, Montbonnot-Saint-Martin, France) at 6800 rpm, for two cycles of 20 s on and 30 s off. Subsequently, the crude lysate was added directly to the RPA-reaction mix.

### 2.5. Design of Primers, Probes and Internal Amplification Control 

Primers and probes were designed according to the TwistAmp^®^ instruction manual [67] from TwistDx Limited (Cambridge, UK). The target sequence was a highly conserved region of the *lasB* gene of *P. aeruginosa* [68]. The National Center for Biotechnology Information’s (NCBI) Blast tool [69] and primer-blast tool [70] were used for primer design, while the specific nfo-probe was adapted manually. Free energy calculations and secondary structure predictions were performed using the software OligoPAD (Version 0.3.0.2, GNWI mbH, Dortmund, Germany). Primer and probe sequences are indicated in Table 1. The set of *lasB* specific primers (*lasB*-fwd and *lasB*-rev primer) and a matching probe (*lasB*-probe) give rise to a single-labeled (digoxigenin) 161 bp product and a double-labeled (digoxigenin and biotin) 123 bp product. Only the double-labeled product can be detected via lateral flow assay.

**Figure 1:**
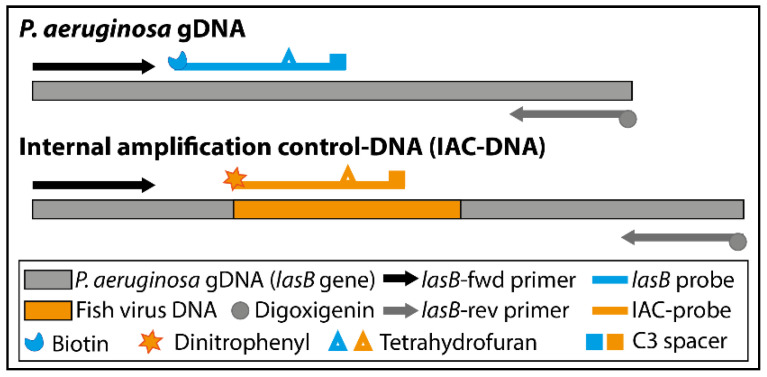
RPA assay schematic. The RPA assay targets two DNA sequences: A sequence within the *P. aeruginosa* genome (*lasB* gene) and an amplification control (IAC)-DNA sequence.

Furthermore, a competitive internal amplification control (IAC) was designed to exclude false negative results (see Figure 1). The practical considerations of IAC design for diagnostic applications are well discussed by Hoorfar et al. [11]. The IAC-DNA consisted of a 61 bp fragment of a coding region of the hemorrhagic septicemia virus from rainbow trout (fish virus DNA) (accession no. X66134) and is flanked by *lasB* primer binding sites. The same primer pair (*lasB*-fwd primer and *lasB*-rev primer) was used to amplify the target DNA (*lasB*, *P. aeruginosa*) and the IAC-DNA. A specific probe (IAC-probe) was used to hybridize to the fish virus DNA sequence of the IAC-DNA. Probe and primers were again checked for specificity, dimer and hairpin formation. The set of primers and IAC-DNA specific probe (IAC-probe) produce a single-labeled (digoxigenin) 192 bp product and a double-labeled (digoxigenin and DNP) 138 bp product.

To determine the specificity of primers and probes, the gDNA of the respective microorganisms mentioned above was isolated via DNeasy PowerLyzer Microbial Kit from Qiagen GmbH (Hilden, Germany) and stored at −20 °C until use. Figure S2 (see supplementary data) indicates that primers (*lasB*-fwd primer and *lasB*-rev primer) and probes (*lasB* probe and IAC-probe) were binding specifically to their target and showed no cross-reaction with other microorganisms.

### 2.6. Synthesis of Antibody-Conjugated Fluorescence Microspheres

The sheep anti-digoxigenin antibody was conjugated to 0.2 µm sized carboxylate-modified microspheres using EDC/NHS chemistry. Therefore, 7 µg antibody was dissolved in MES buffer (50 mM, pH 5.5). Next, 100 µg of the microspheres and 2 µg EDC and 2 µg NHS were added. The suspension was adjusted to a final volume of 400 µL and incubated on a rotary mixer at room temperature for 2 h. To quench the reaction, 300 µg glycine was added, and the reaction mix was incubated for another 30 min. After centrifugation for 8 min at 14,000 rpm the anti-digoxigenin-conjugated microspheres were washed once with storage buffer (1× PBS containing 0.05% (*v/v*) Tween 20, 0.5% (*w/v*) biotin-free BSA). Finally, the functionalized beads were resuspended in 100 µL storage buffer (to yield 0.1% bead solid) and stored at 4 °C until use.

### 2.7. Assembly of the Lateral Flow Dipstick

For the fabrication of lateral flow dipsticks, the backing card (4 × 30 cm), nitrocellulose membrane (2 × 30 cm) and absorbent pad (2.2 × 30 cm) were assembled accordingly. Polystreptavidin (75 µg/mL), anti-DNP antibody (300 µg/mL), and donkey anti-sheep IgG antibody (500 µg/mL) were diluted in PBS containing 0.1% (*w/v*) biotin-free BSA and 1% (*w/v*) trehalose. The solutions were printed (1 µL/cm) onto the nitrocellulose membrane with a line-to-line distance of 2.5 mm using a line printer (AD3220TM Aspirate/Dispense Platform, BioDot Limited, Chichester, United Kingdom). Polystreptavidin binds the double-labeled (biotin, digoxigenin) target DNA amplicon and thus represents the test line (TL). The anti-DNP antibody binds the double-labeled (DNP, digoxigenin) IAC-DNA amplicon and thus represents the internal amplification control line (IAC). The sheep anti-digoxigenin-conjugated fluorescence microspheres bind to the anti-sheep IgG antibody which therefore acts as flow control line (FC). The lateral flow dipstick sheets were dried for at least 24 h at room temperature (RT) and subsequently cut into lateral flow dipsticks with a width of 4.4 mm using a guillotine cutter (A-Point Guillotine Cutter, Arista Biologicals Inc., Allentown, PA, USA). The lateral flow dipsticks were stored at RT in a box containing silica gel until use. 

### 2.8. RPA-Lateral Flow Assay Procedure

The RPA was performed in a 50 µL volume using the TwistAmp^®^ nfo kit (TwistDx limited, Cambridge, UK). Briefly, 29.5 µL 1× rehydration buffer was mixed with 1.25 µL *lasB*-fwd (10 µM), 1.25 µL *lasB*-rev (10 µM), 1.2 µL *lasB* probe (10 µM), 1.2 µL IAC-Probe (10 µM), and 2300 copies of IAC-DNA per reaction. Subsequently, 1 µL of gDNA and 11.1 µL ddH2O or 5 µL crude lysate and 7.1 µL ddH2O were added to the reaction mix. Next, the RPA reaction pellet and 2.5 µL of magnesium acetate (280 nM) were added and the reaction was incubated for 20 min at 37 °C. Optimization of the RPA reaction regarding primer, probe and IAC-DNA concentration is shown in Figures S3 and S4. 

Subsequently, the RPA reaction was diluted 1:30 in 50 µL running buffer containing 120 µg/mL anti-digoxigenin-conjugated microspheres, 0.5% (*w/v*) biotin-free BSA, and 0.1% (*v/v*) Tween 20. The lateral flow strip was dipped into the solution and incubated for 7 min at RT and imaged in a fluorescence microscope (Lionheart LX Automated Microscope, BioTek Instruments Inc., Bad Friedrichshall, Germany). Fluorescence intensity of test line, IAC, and FC was determined by image analysis using ImageJ (Fiji is just ImageJ) [71,72].

### 2.9. Detecting P. aeruginosa in Spiked Wound Exudate or Buffer

The utility of the streamlined protocol for pathogen detection from human wound exudate was evaluated. Therefore, PBS or wound exudate from non-infected patients was spiked with different concentrations of *P. aeruginosa* (1.5 ± 0.4 × 10^4^–1.5 ± 0.4 × 10^7^ CFU/mL). For specificity validation experiments, PBS was spiked with one of the following microorganisms: *S. aureus*, *S. epidermidis*, *S. agalactiae*, *E. coli*, *K. pneumoniae*, *E. faecalis*, and *P. mirabilis*. Subsequently, the microorganisms were lysed via bead beating, crude lysate was added to the RPA reaction mix, and the amplification products were detected via lateral flow assay.

### 2.10. Statistical Analysis

All measurements were conducted three times per experiment, and all experiments were performed in triplicates. Statistical analysis was performed using Origin (OriginLab Corporation, Northampton, MA, USA). A four-parameter logistic non-linear regression model (PL4) was used for curve fitting analysis (see supplementary data, Tables S1 and S2). The LOD was calculated from the mean fluorescence intensity (y) and standard deviation (SD) of the blank and of a low concentration sample [73]:

y_LOD_ = (y_blank_ + 1.645 * SD_blank_) + 1.645 * SD_low concentration sample_

The corresponding concentration and confidence interval was then calculated by interpolating the calculated fluorescence intensity of the LOD (y_LOD_) into the sigmoidal fit curve equation.

## 3. Results

### 3.1. Principle of the Paper-Based Approach for Pathogen Detection in Wound Exudate

**Figure 2:**
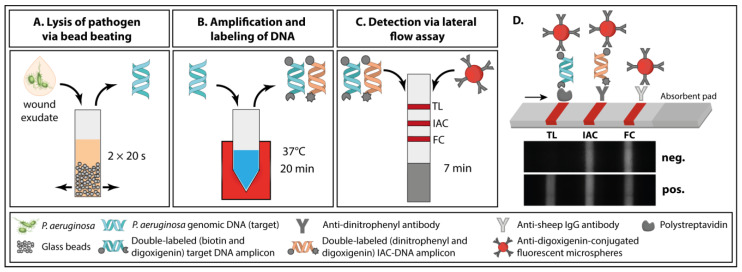
Principle of the paper-based approach for *P. aeruginosa* detection in wound exudate. (**A**) Wound exudate spiked with *P. aeruginosa* is transferred into a top-screw tube containing glass beads and the pathogen is lysed via bead beating. (**B**) Then, the crude lysate is transferred to the RPA reaction. Target DNA and IAC-DNA are amplified and double-labeled target DNA amplicons and double-labeled IAC-DNA amplicons are generated. (**C**) Anti-digoxigenin-conjugate fluorescent beads are binding to the amplicons and they are detected via lateral flow assay. (**D**) Schematic drawing of the NALFIA. The double-labeled target DNA amplicon binds to the test line (TL) leading to a positive test result. The double-labeled IAC-DNA amplicon binds to a separate control line (IAC) to exclude false negative results, and a flow control (FC) shows whether the sample was processed.

The developed streamlined protocol for pathogen identification in wound exudate combines all three necessary steps (Figure 2): (A) lysis of the pathogen via bead beating, (B) amplification and generation of double-labeled amplicons from crude lysate via RPA, and (C) detection of the double-labeled amplification products via NALFIA. 

Our approach has a sample-to-result turnaround time of less than 30 min with only three hands-on steps (see Figure 2): The user adds the wound exudate to the lysis tube containing glass beads (1st hands-on step). After bead beating, the crude lysate is transferred directly to the already prepared RPA reaction and the DNA is amplified and labeled within 20 min (2nd hands-on step). Subsequently, the user dilutes the amplification reaction with running buffer and dips the lateral flow strip into the reaction (3rd hands-on step). After 7 min, the NALFIA can be analyzed via commercial lateral flow reader.

**Figure 3:**
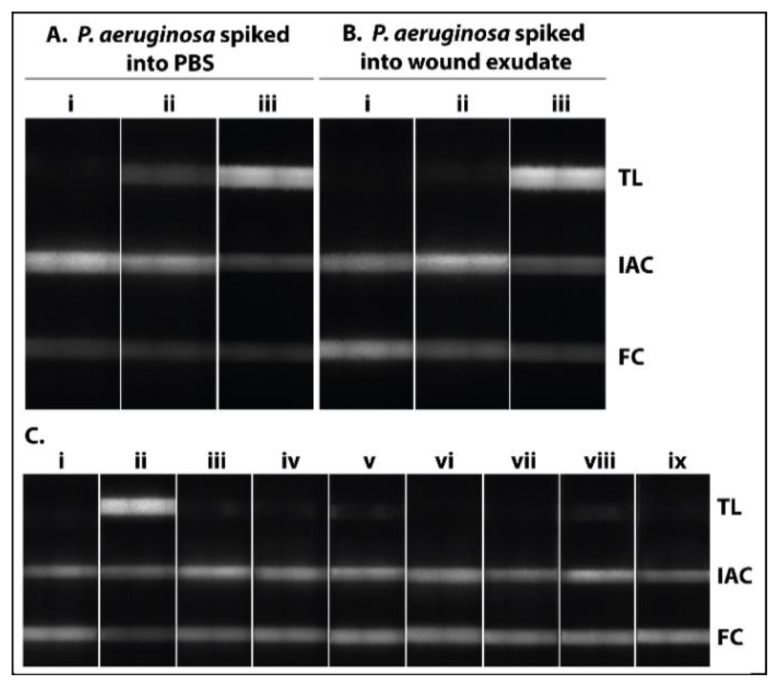
Lysis control and specificity of the paper-based approach for *P. aeruginosa* detection. *P. aeruginosa* was spiked into (**A**) PBS or **(B**) wound exudate, whereas the negative control (i) contains no bacteria. The spiked samples were added without (ii) or after bead beating (2 × 20 s (iii) to the RPA reaction and the amplification products were detected via NALFIA. The NALFIA showed a clear signal at the test line (TL) for the lysed sample. Only a weak signal was observed for the sample that was not lysed. (**C**) Specificity of the paper-based approach for the detection of *P. aeruginosa* over other pathogens present in wounds. The following pathogens were spiked into PBS: Without bacteria (i), *P. aeruginosa* (ii), *S. aureus* (iii), *S. epidermidis* (iv), *S. agalactiae* (v), *E. coli* (vi), *K. pneumoniae* (vii), *E. faecalis* (viii), *P. mirabilis* (ix). The samples were lysed via bead beating, and the lysate was added directly to the RPA reaction. Subsequently, the amplification products were detected via NALFIA. The test line (TL) showed a positive signal for the sample spiked with *P. aeruginosa*, whereas the signals for the other pathogens were below the fluorescence intensity of the detection limit. Signals for internal amplification control (IAC) and flow control (FC) were observed for all NALFIA strips. The experiments were conducted three times and were performed in triplicates.

We selected bead beating (Figure 2A) as a fast, effective and simple way to directly lyse pathogens in wound exudate. This mechanical disruption technique can be ubiquitously applied for different microorganisms regardless of their nature and the sample matrix [74,75]. In this study, two cycles of 20 s at 6800 rpm and a break of 30 s were sufficient to lyse the whole pathogen panel used for specificity investigations (see Figure 3 and supplementary material Figures S5 and S6). Without any need for purification after lysis, we added the crude lysate directly to the RPA-reaction mix (Figure 2B).

The target DNA was amplified and labeled at 37 °C within 20 min. The addition of a specific probe increases the specificity of the amplification method. The probe contains a polymerase extension blocking group (C3 spacer) and a tetrahydrofuran (THF) residue, which is cleaved by a nfo nuclease, but only when the probe hybridizes to its target sequence.

Cleavage removes the blocking group and transforms the probe into a new primer. Thus, two amplification products can be observed on the agarose gel (see supplementary data Figure S2): a single- and a double-labeled amplification product. However, only the double-labeled amplicon is detected via NALFIA. To exclude false negative results, we designed an IAC-DNA that is co-amplified with the target DNA. We decided to use a competitive IAC strategy. This means the same set of primer is used to amplify target and IAC-DNA. The use of a specific IAC-probe allows the separate detection of the IAC-DNA amplicon. The IAC-DNA concentration is the most critical parameter and needs to be optimized to the lowest concentration to yield reproducible DNA amplification [11]. A minimum of 2300 copies per reaction was required for a clear IAC signal when using wound exudate (see supplementary material, Figure S4).

The combination of RPA with NALFIA is the ideal choice for the qualitative detection of pathogens at the POC. In the presence of double-labeled target DNA amplicons, a signal is generated at the test line which constitutes a positive test result (Figure 2D). The double-labeled IAC-DNA amplicon binds to a separate control line, to exclude false negative results that might result from RPA inhibition. A flow control (FC) shows whether the sample has passed through the lateral flow strip appropriately.

We used fluorescent microspheres as detecting label since they are described to enhance the detection signal and improve the sensitivity of LFA due to their configurational stability and high photostability. The LFA can be analyzed by commercially available lateral flow readers that eliminate subjective test result interpretation [76,77,78].

The approach described herein integrates sample preparation, sample processing and detection into a fast and straightforward streamlined protocol with only three simple hands-on steps and a sample-to-result turnaround time of less than 30 min.

### 3.2. Analytical Performance of the Paper-Based Approach for Pathogen Detection

To confirm that the lysis procedure is successful, we spiked PBS (Figure 3A) and wound exudate (Figure 3B) with 1.5 ± 0.4 × 10^7^ CFU/mL *P. aeruginosa* and added the bacterial suspension, with and without bead beating, to the RPA reaction mix. As shown in Figure 3A,B a clear difference was observed between samples that have been lysed (iii) or not (ii). To investigate the analytical specificity of the approach, *P. aeruginosa* and a panel of other pathogens (*S. aureus*, *S. epidermidis*, *S. agalactiae*, *E. coli*, *K. pneumoniae*, *E. faecalis*, *P. mirabilis*) associated with wound infection were spiked into PBS (1:10 dilution of bacterial suspension at a OD_600_ of 0.36). After lysis and amplification, the amplification products were detected via NALFIA. A clear signal was observed at the test line for *P. aeruginosa*, whereas the signals for the other pathogens were below the fluorescence intensity of the detection limit, corresponding to a cutoff of 18,256 relative fluorescence units (RFU) (Figure 3C). The IAC and FC signals were positive for all tests, indicating a valid result. Thus, no cross-reaction with other pathogens was observed.

**Figure 4:**
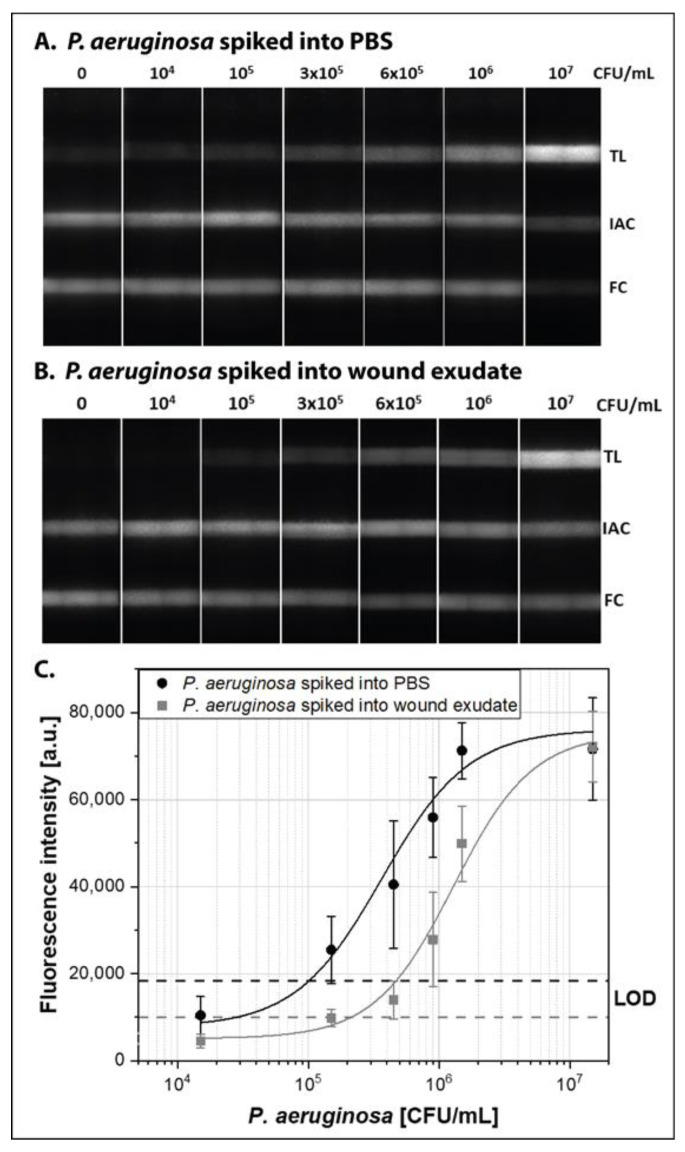
Analytical sensitivity of the paper-based approach for the detection of *P. aeruginosa*. PBS (**A**) and wound exudate (**B**) was spiked with 1.5 ± 0.4 × 10^4^–1.5 ± 0.4 × 10^7^ CFU/mL of *P. aeruginosa*. The samples were lysed via bead beating and the crude lysate was added to the amplification reaction. The presence of double-labeled target DNA amplicons generated a signal at the test line (TL) of the NALFIA. The internal amplification control (IAC) and flow control (FC) signal was positive for all tests, indicating a valid result. (**C**) To determine the limit of detection (LOD), the fluorescence intensity of the TL was determined via ImageJ and a sigmoidal fit curve was generated for the detection of *P. aeruginosa* in PBS (black line) and wound exudate (grey line). The dashed lines represent the fluorescence intensity of the LOD in PBS (black dashed line) and wound exudate (grey dashed line). The error bars indicate one standard deviation. The experiments were conducted three times and were performed in triplicates.

To determine the analytical sensitivity of the approach, we spiked 1.5 ± 0.4 × 10^4^–1.5 ± 0.4 × 10^7^ CFU/mL of *P. aeruginosa* into PBS and lysed them via bead beating. The crude lysate was added directly to the RPA reaction and the resulting amplification products were detected via NALFIA (Figure 4A). A fluorescence intensity of the LOD of 18,356 RFU was calculated (see supplementary data Tables S1 and S2) and therefore a LOD of 1 × 10^5^ CFU/mL (95% confidence interval: 3.7 × 10^4^–2.5 × 10^5^ CFU/mL) was determined. This corresponds to 100 CFU/µL, which is comparable to the detection limit of 121 copies/reaction that was determined for purified genomic DNA (see supplementary data Figure S7). The lateral flow strips of the sensitivity validation experiments are shown in Figure 4A, and the corresponding sigmoidal fit curve and the LOD is shown in Figure 4C. 

### 3.3. Demonstration with P. aeruginosa Spiked Human Wound Exudate

In order to demonstrate the applicability of the approach for the detection of pathogens in wound exudate, we spiked human wound exudate from non-infected patients with *P. aeruginosa*. As shown in Figure 3B, the lysis procedure is also successful for wound exudate. To establish the sensitivity achievable with wound exudate, the bodily fluid was spiked with 1.5 ± 0.4 × 10^4^–1.5 ± 0.4 × 10^7^ CFU/mL *P. aeruginosa*. Again, the crude lysate was added directly to the RPA reaction and the amplification products were detected via NALFIA. Figure 4B,C show the corresponding lateral flow strips and fit curve. We observed a lower fluorescence background in wound exudate (5112 RFU) compared to PBS (6886 RFU). Thus, for the detection of *P. aeruginosa* in wound exudate, a fluorescence intensity of the LOD of 9993 RFU was calculated, which corresponds to 2.1 × 10^5^ CFU/mL (95% confidence interval: 9.6 × 10^4^–4.7 × 10^5^ CFU/mL). Therefore, we show the successful pathogen identification in wound exudate after crude lysis, direct amplification via RPA, and detection via NALFIA.

## 4. Discussion

Currently there are no paper-based POC tests available for the detection of pathogens in wound exudate. However, a rapid test combined with fast sample preparation and processing has enormous potential to improve the clinical outcome of patients with wound infection by enabling an early detection of the pathogen and a target-oriented therapy.

Tedious multi-step lengthy protocols are described in literature to remove contaminants and inhibitors from the clinically relevant, complex samples, which limits the use of NAATs at the POC [79]. To address the need for a reliable rapid test for the detection of pathogens in wound exudate, we combined straightforward sample preparation with RPA. The RPA is a robust amplification method that works in the presence of known PCR inhibitors and facilitates the amplification of nucleic acids from crude extracts [80]. We show the successful pathogen detection from wound exudate after crude lysis and direct amplification via RPA.

In addition, a competitive IAC was integrated that excludes false negative results and, in combination with the flow control, ensures the validity of the test result. Until now only a part of the published RPA approaches incorporated an IAC [46,81,82,83,84,85,86,87]. The main advantages over the non-competitive method are: (1) avoiding the risk of undesired interaction of multiple primers and (2) a more accurate reflection of the amplification, due to the identical primers and reaction conditions. In this regard, it is important to keep the IAC-DNA concentration at the lowest concentration that yields reproducible DNA amplification [11]. We determined a minimum of 2300 copies IAC-DNA per reaction when using wound exudate and thus, successfully integrated the IAC into the streamlined protocol.

As mentioned above it is believed that a microbial load of >10^5^ CFU per mL of wound exudate is required to reach a stage of local infection (critical colonization). At this time point, clinical intervention becomes necessary [63,64,65,66,88]. A qualitative rapid test for the detection of pathogens in wound exudate with a LOD of 10^5^ CFU/mL offers a fast decision tool to (1) decide if a wound is infected and (2) start a target-oriented therapy using specific antibiotics and antibacterial substances. We determined a LOD of 1 × 10^5^ CFU/mL in PBS and a LOD of 2.1 × 10^5^ CFU/mL in wound exudate. Thus, the sensitivity of our approach was slightly reduced for using wound exudate as a sample matrix, compared to the detection of *P. aeruginosa* in PBS. The biological matrix might influence the amplification efficiency and thus lead to the differences in the LOD. The addition of amplification enhancers such as betaine or trehalose might improve the amplification reaction [89].

Thus, we show the successful pathogen detection in wound exudate after crude lysis, direct amplification via RPA, and detection via NALFIA in a clinical relevant range. However, remnants of a treated bacterial infection might lead to a false-positive test result. In general, this limitation is concerning all amplification methods. Thus, it is important to consider the test result together with the clinical appearance of the wound. A future test combining the detection of the pathogen together with biomarkers of the host immune response could indicate a successfully treated bacterial infection. Additionally, we selected an approach that can be analyzed by commercially available lateral flow readers that eliminate subjective test result interpretation and provides traceability by automatically recording the test data. Thus, a reader facilitates the (future) multiplex detection of pathogens associated with wound infection [76,77,78].

Therefore, our research work significantly exceeds the current state of the art by (1) implementing an easy workflow for the detection of pathogens in wound exudate with a turn-around time of 30 min, and (2) showing a straightforward approach covering for the first time the complete analytical process chain from sample to answer with only three simple hands-on steps. Controls ensure the validity of the test result and, importantly, no cross-reaction was observed with other pathogens associated with wound infection. In future, our focus will be on integrating all fundamental operation steps into one single paper-based device. In this manner, the rapid test should fulfill POC requirements such as a closed cartridge (to minimize the risk of amplicon carryover contaminations) or a sample-to-device interface, and should expand the panel for multiplex pathogen detection in wound exudate [13,90,91]. Thus, this rapid test paves the way towards reliable pathogen detection for a target-oriented therapy of infected wounds at the POC.

## Figures and Tables

**Table 1:** Primers, probes and internal amplification control-DNA sequences.

Name	Sequence (5′-3′)
*lasB*-fwd primer	GAGAATGACAAAGTGGAACTGGTGATCCGCCTG
*lasB*-rev primer	Dig-GCCAGGCCTTCCCACTGATCGAGCACTTCGCCG
*lasB* probe	Biotin-GAACAACATCGCCCAACTGGTCTA CAACGT[H]TCCTACCTGATTCCC-C3 spacer
IAC-probe	DNP-CAACTGCAGGGACGATTCCTTTGTCC CGAT[H]CGACCAGCTCAACTC-C3 spacer
IAC-DNA	AAGACCGAGAATGACAAAGTGGAACTGGTGATCCGCCTGGGCGATATACACTCATCCCTCCAACTGCAGGGACGATTCCTTTGTCCCGATTCGACCAGCTCAACTCAGGTGTCCTCATGAAGGCGAGGGACTGTCGCGGCCGCATTTCGTCATCGACGCCAAGACCGGCGAAGTGCTCGATCAGTGGGAAGGCCTGGCCCACGC

Dig, digoxigenin; H, tetrahydrofuran; C3 spacer, polymerase extension blocking group; DNP, dinitrophenyl; IAC-DNA, internal amplification control-DNA; underlined sequence, fish virus DNA sequence.

## Data Availability

Not applicable.

## Supplementary Materials

The following are available online at https://www.mdpi.com/2079-6374/11/3/74/s1, Figure S1: Determination of the OD_600_ to CFU per mL correlation., Figure S2: Determination of the analytical specificity of the RPA using isolated gDNA of *P. aeruginosa* and other pathogens present in infected wounds., Figure S3: Optimization of the RPA reaction regarding primer and probe concentration., Figure S4: Optimization of the IAC-DNA concentration for the paper-based detection of *P. aeruginosa* in wound exudate., Figure S5: Optimization of the lysis protocol., Figure S6: CFU counting before and after bead beating for the whole microorganism panel., Figure S7: Determination of the analytical sensitivity of the RPA using isolated gDNA of *P. aeruginosa*., Table S1: Sigmoidal fit curve analysis and linear regression line analysis., Table S2: Mean, standard deviation (SD) and coefficient of variation (CV).
