# Supplementary material for: Rapid Detection of Pathogens in Wound Exudate via Nucleic Acid Lateral Flow Immunoassay
Source: Biosensors (Basel). 2021 Mar 6;11(3):74. doi: 10.3390/bios11030074 (PMC8035659; doi:10.3390/bios11030074)
Supplement: Supplementary file 1 [file biosensors-11-00074-s001.pdf]

# Supporting information

# Rapid Detection of Pathogens in Wound Exudate via Nucleic Acid Lateral Flow Immunoassay

**Anna Brunauer [1], René D. Verboket [2], Daniel M. Kainz [1,3], Felix von Stetten [1,3] and Susanna M. Früh [1,3,*]**

[1] Laboratory for MEMS Applications, IMTEK—Department of Microsystems Engineering, University of
Freiburg, Georges-Koehler-Allee 103, 79110 Freiburg, Germany;
anna.brunauer@imtek.uni-freiburg.de (A.B.); daniel.kainz@imtek.uni-freiburg.de (D.M.K.); felix.von.stetten@hahn-schickard.de (F.v.S.)
[2] Department of Trauma-, Hand- and Reconstructive Surgery, University Hospital Frankfurt,
Johann Wolfgang Goethe University, Theodor-Stern-Kai 7, 60590 Frankfurt am Main, Germany;
Rene.Verboket@kgu.de
[3] Hahn-Schickard, Georges-Koehler-Allee 103, 79110 Freiburg, Germany
* Correspondence: Susanna.Frueh@Hahn-Schickard.de; Tel.: +49-761-203-73209

**Table of Content:**

**Figure S1.** Determination of the $OD_{600}$ to CFU per ml correlation.

**Figure S2.** Determination of the analytical specificity of the RPA using isolated gDNA of *P. aeruginosa* and other pathogens present in infected wounds.

**Figure S3.** Optimization of the RPA reaction regarding primer and probe concentration.

**Figure S4.** Optimization of the IAC-DNA concentration for the paper-based detection of *P. aeruginosa* in wound exudate.

**Figure S5.** Optimization of the lysis protocol.

**Figure S6.** CFU counting before and after bead beating for the whole microorganism panel.

**Figure S7.** Determination of the analytical sensitivity of the RPA using isolated gDNA of *P. aeruginosa*.

**Table S1.** Sigmoidal fit curve analysis and linear regression line analysis.

**Table S2.** Mean, standard deviation (SD), coefficient of variation (CV) and limit of detection (LOD).

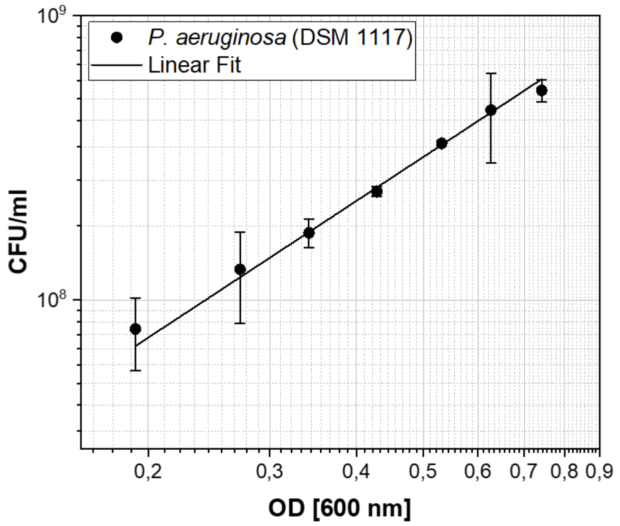


**Figure S1.** Determination of the $OD_{600}$ to CFU per ml correlation. The $OD_{600}$ of a *P. aeruginosa* culture was measured every 30 min for 6.5 hours. In addition, serial dilutions of the culture were plated on LB agar plates to determine the corresponding CFU per ml for each measurement. A linear regression analysis was used to determine the relationship between $OD_{600}$ and CFU per ml (see Table S1). The experiment was conducted two times independently, and all measurements were performed in triplicates.

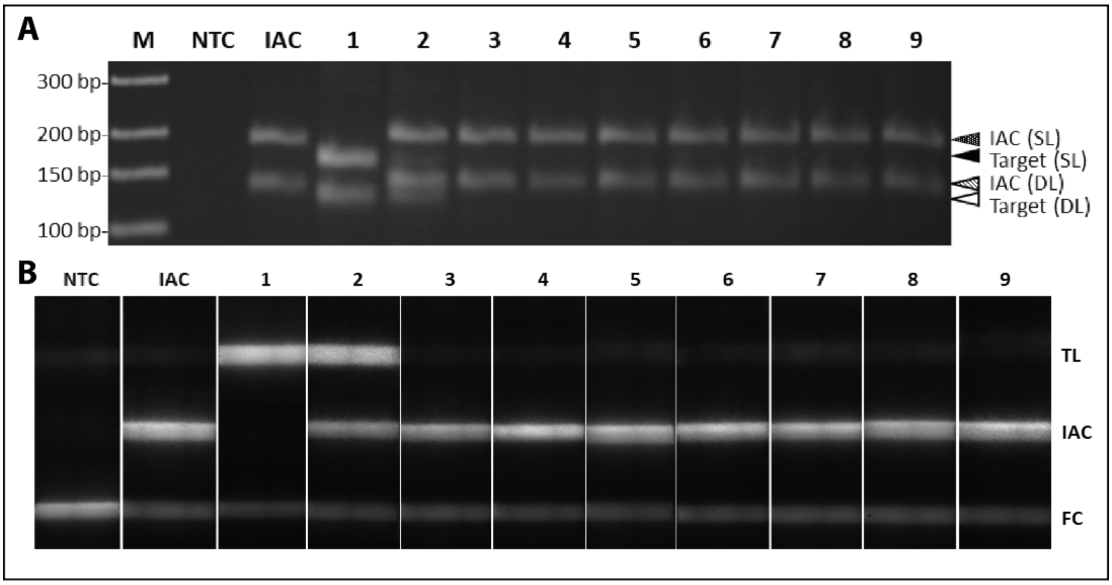


**Figure S2.** Determination of the analytical specificity of the RPA using isolated gDNA of *P. aeruginosa* and other pathogens present in infected wounds. (A) Agarose gel showing the specific binding of primers and probes to 106 copies of target DNA (1) and $2.3x10^3$ copies of IAC-DNA (IAC). Amplification of target DNA leads to a 161 bp single-labelled (SL) amplification product and a 123 bp double-labelled (DL) amplification product. Additionally, the amplification of the IAC-DNA leads to a 192 bp SL amplification product and a 138 bp DL amplification product. Sample 2 contains both 104 copies of target DNA and $2.3x10^3$ copies of IAC-DNA; therefore, four different bands can be observed. No bands were observed for reactions without target DNA and IAC-DNA (no template control, NTC). Samples 3 to 9 contain $2.3x10^3$ copies of IAC-DNA in combination with purified gDNA of one of the following bacteria: *S. aureus* (3), *S. epidermidis* (4), *S. agalactiae* (5), *E. coli* (6), *K. pneumoniae* (7), *E. faecalis* (8). *P. mirabilis* (9), respectively. Only the two bands of the IAC-DNA amplification products are present. (B) The same samples were detected via NALFIA confirming the agarose-gel electrophoresis result. The double-labeled target DNA amplicon binds to the test line (TL) whereas the double-labelled IAC-DNA amplicon binds to a separate line

(IAC) and confirming together with flow control (FC) the validity of the result. The experiment was conducted three times and was performed in triplicates.

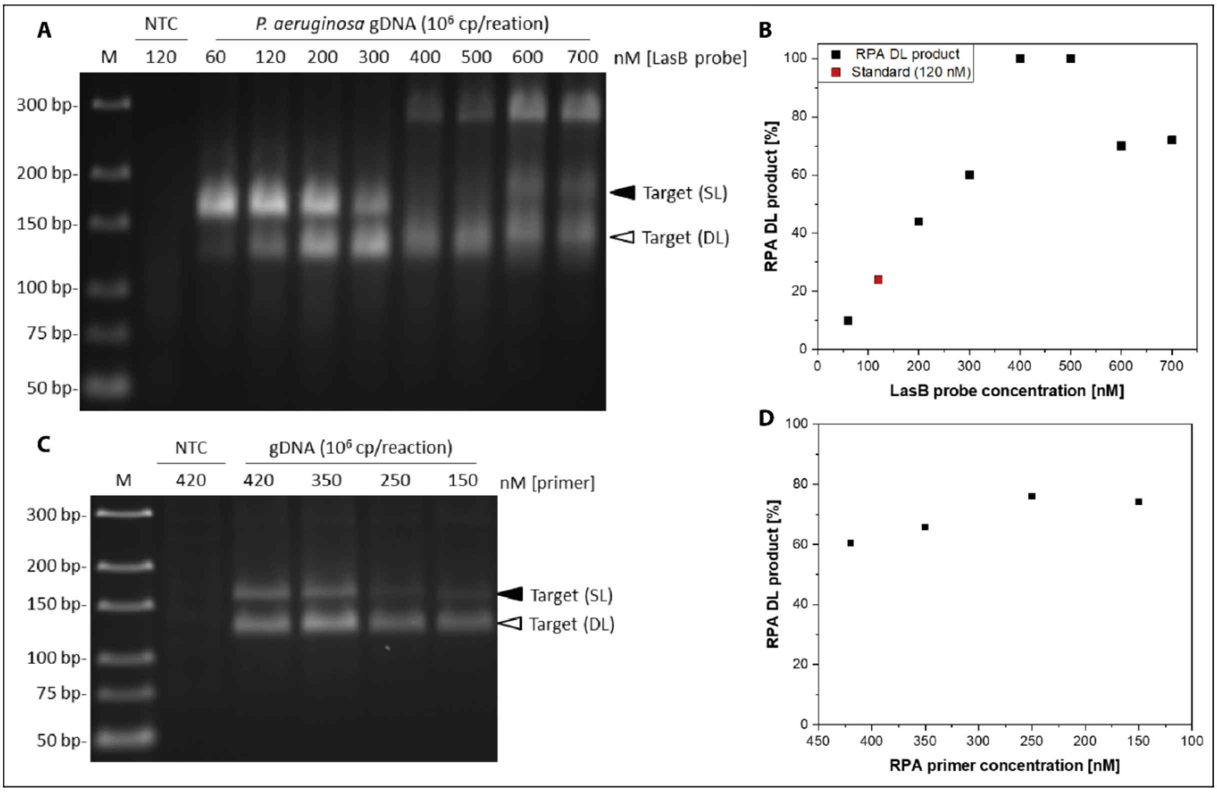


**Figure S3.** Optimization of the RPA reaction regarding primer and probe concentration. The set of *lasB* specific primers (*lasB*-fwd and *lasB*-rev primer) and probe (*lasB* probe) produce a single-labeled (SL) 161 bp product and a double-labeled (DL) 123 bp product. Only the double-labeled product can be detected via lateral flow assay. Thus, we optimized the reaction regarding primer and probe concentration to increase the percentage of DL product. Different *lasB* probe concentrations (60 – 700 nM) were added to the RPA reaction containing 420 nM of *lasB*-fwd primer and 420 nM of *lasB*-rev primer. The amplification products were analyzed via agarose gel electrophoresis (A), and the ratio between SL and DL product was determined via ImageJ (B). A *lasB* probe concentration >300 nM leaded to unspecific products (band at about 300 bp). The highest percentage of DL product was achieved with 200 nM and 300 nM of lasB probe. Subsequently, we optimized the primer concentration by adding different *lasB*-fwd and *lasB*-rev primer concentration (150 – 420 nM) to the RPA reaction containing 240 nM *lasB* probe (C, D). The highest percentage of DL product was achieved with 250 nM of *lasB*-fwd and *lasB*-rev primer. Thus, the optimized RPA reaction contained 250 nM of each primer and 240 nM of the *lasB* primer.

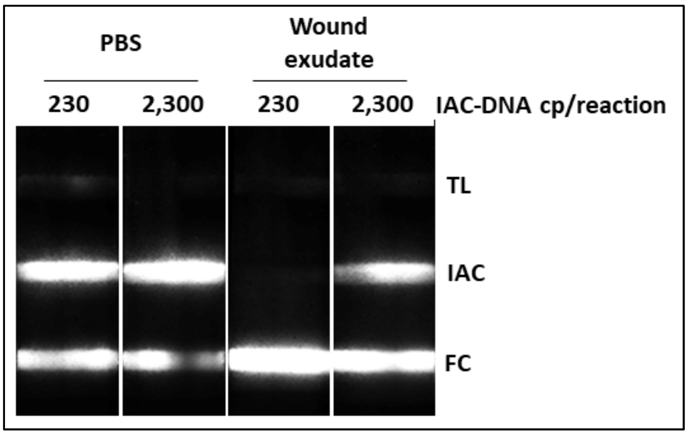

**Figure S4.** Optimization of the IAC-DNA concentration for the paper-based detection of *P. aeruginosa* in wound exudate. We used a competitive design, which means that the same set of primer is used to amplify target and IAC-DNA. The IAC-DNA concentration is the most critical parameter and needs to be optimized to the lowest reproducible concentration. A minimum of 2,300 copies per reaction was required for a clear IAC signal when wound exudate lysate was used. In contrast, 230 copies of IAC-DNA were sufficient for the amplification in buffer.

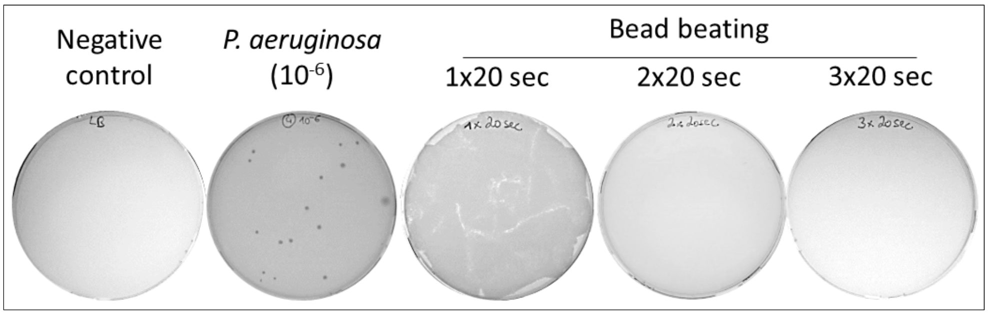


**Figure S5.** Optimization of the lysis protocol. To determine the optimal protocol for the lysis of *P. aeruginosa* via Precellys® bead-beating homogenizer (Bertin Technologies), the pathogen was spiked into PBS ($1.5 \pm 0.4 \times 10^7$ CFU/ml) and lysed at 6,800 rpm for one cycle of 20 seconds on (1x20 sec), two cycles of 20 seconds on and 30 seconds off (2x20 sec), or three cycles of 20 second on and 30 seconds off (3x20 sec). No colonies were observed for the negative control (LB diluted 1:10 in PBS), whereas microbial growth was observed for samples that were not lysed via bead beating (*P. aeruginosa* ($10^{-6}$)). Microbial growth was also observed for 1x20 sec (crude lysate, no dilution), whereas no colonies were observed for 2x20 sec and 3x20 sec (crude lysate, no dilution). For all further experiments, the 2x30 sec protocol was used. The experiment was conducted twice and was performed in triplicates.

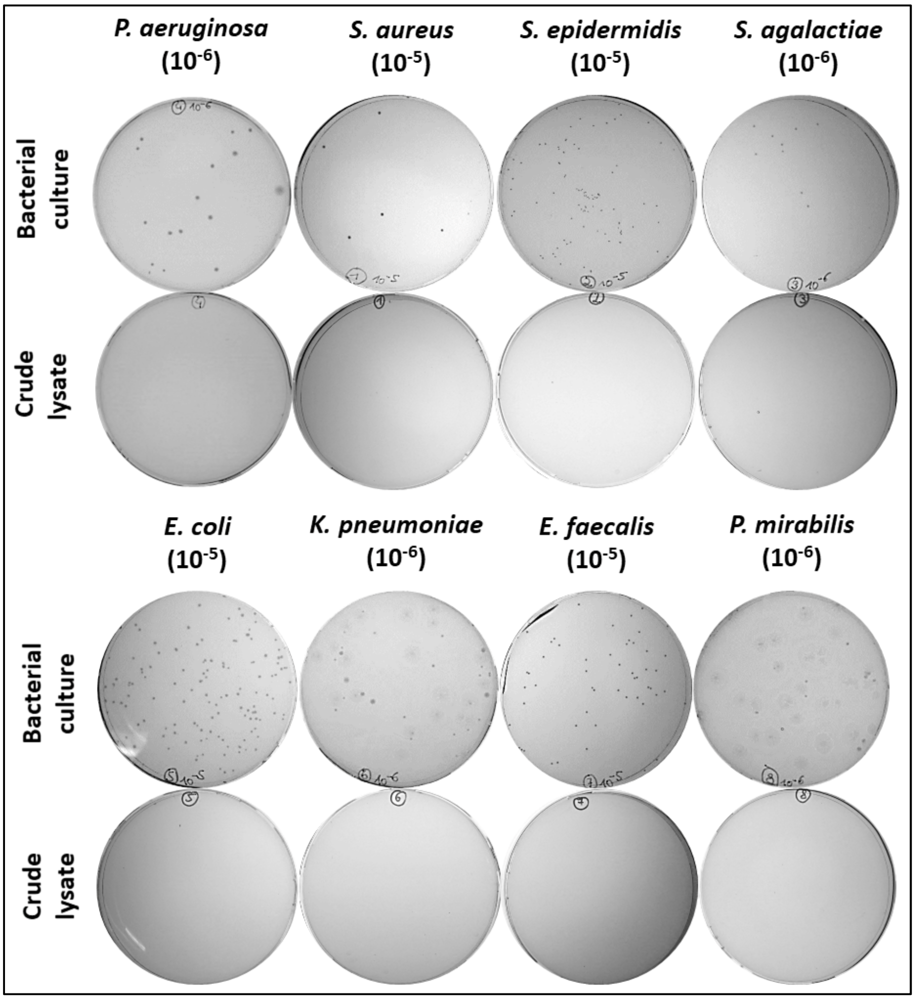

**Figure S6.** CFU counting before and after bead beating for the whole microorganism panel. PBS was spiked with *P. aeruginosa* or one of the following pathogens associated with infected wounds (1:10 dilution of bacterial suspension at a $OD_{600}$ of 0.36): *S. aureus*, *S. epidermidis*, *S. agalactiae*, *E. coli*, *K. pneumoniae*, *E. faecalis*, or *P. mirabilis*. The concentration of the bacteria before lysis was determined via serial dilution plating on LB agar plates, whereas 100 µl of the crude lysate, without dilution, was used to determine the lysis efficiency. Before lysis, the following amount of bacteria was present: $2.3 \pm 0.1 \times 10^7$ CFU/ml *P. aeruginosa*, $3.4 \pm 2.3 \times 10^6$ CFU/ml *S. aureus*, $9.4 \pm 1.6 \times 10^6$ CFU/ml *S. epidermidis*, $1.1 \pm 0.1 \times 10^7$ CFU/ml *S. agalactiae*, $1.2 \pm 0.3 \times 10^7$ CFU/ml *E. coli*, $4.1 \pm 1.6 \times 10^7$ CFU/ml *K. pneumoniae*, $5.9 \pm 1.8 \times 10^6$ CFU/ml *E. faecalis*, and $4.9 \pm 1.4 \times 10^7$ CFU/ml *P. mirabilis*. No colonies were observed after bead beating at 6,800 rpm for two cycles of 20 seconds on and 30 seconds off for the whole microorganism panel. The experiment was conducted three times and was performed in triplicates.

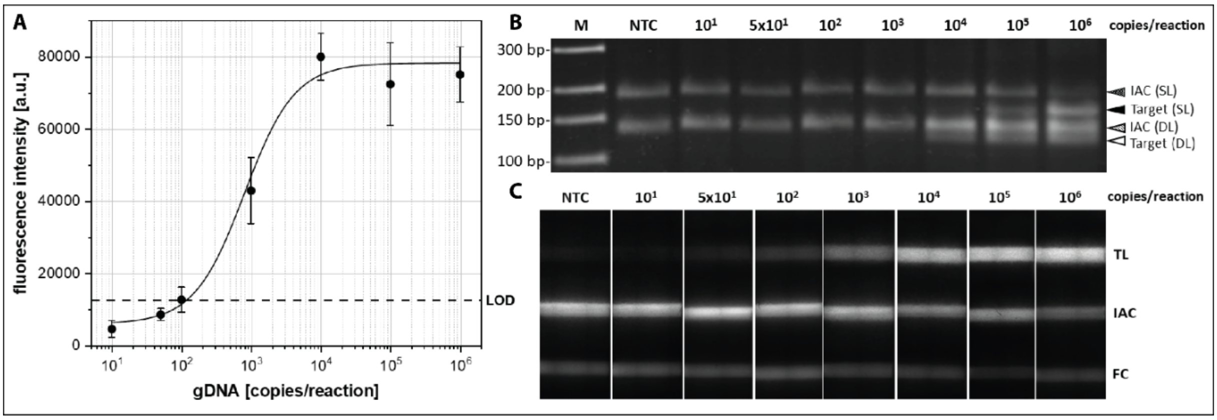


**Figure S7.** Determination of the analytical sensitivity of the RPA using isolated gDNA of *P. aeruginosa*. To determine the sensitivity of the RPA reaction, we added $10^1$-$10^6$ copies of *P. aeruginosa* gDNA and $2.3 \times 10^3$ copies of IAC-DNA per reaction. The products of the RPA reaction were analyzed via agarose gel electrophoresis (B) and NALFIA (C). Amplification of target DNA leads to a 161 bp single-labelled (SL) amplification product and a 123 bp double-labelled (DL) amplification product. Moreover, the amplification of the IAC-DNA leads to a 192 bp SL amplification product and a 138 bp DL amplification product. The NTC (no template control) only contains $2.3 \times 10^3$ copies of IAC-DNA. The double-labeled target DNA amplicon binds to the test line (TL) of the NALFIA, whereas the double-labeled IAC-DNA amplicon binds to a separate line (IAC) and confirms (together with FC) the validity of the result. To determine the limit of detection (LOD), the fluorescence intensity of the TL was determined via ImageJ and a sigmoidal fit curve was generated (see Table S1). A LOD of 121 copies gDNA per reaction was achieved. The experiment was conducted three times and was performed in triplicates. The sensitivity of the RPA reaction might be improved by increasing the reaction time and reducing the IAC-DNA concentration to a minimum.

**Table S1.** Sigmoidal fit curve analysis and linear regression line analysis.

| **Sigmoidal fit curve analysis (Figure 4C, PBS)** | |
|---|---|
| **Function** | y = A2 + (A1-A2)/(1 + (x/x0)^p)<br>A1 = 7705.34692 ± 1539.8046<br>A2 = 76125.87622 ± 7537.61567<br>x0 = 367122.51992 ± 115703.29364<br>p = 1.31187 ± 0.36293 |
| **R2** | 0.99034 |
| **Sigmoidal fit curve analysis (Figure 4C, wound exudate)** | |

| | |
|---|---|
| **Function** | y = A2 + (A1-A2)/(1 + (x/x0)^p)<br>A1 = 5067.9743 ± 907.39825<br>A2 = 75209.21871 ± 8269.27905<br>x0 = 1292874.24172 ± 394950.34542<br>p = 1.43436 ± 0.34487 |
| **R2** | 0.97797 |
| **Linear regression line analysis (Figure S1)** | |
| **Function** | y = a + b*x<br>a = 8.98299 ± 0.02508<br>b = 1.59655 ± 0.08378 |
| **R2** | 0.98642 |
| **Sigmoidal fit curve analysis (Figure S7A)** | |
| **Function** | y = A2 + (A1-A2)/(1 + (x/x0)^p)<br>A1 = 5925.54449 ± 916.4034<br>A2 = 78231.50009 ± 3786.87044<br>x0 = 801.79891 ± 245.62217<br>p = 1.20523 ± 0.21427 |
| **R2** | 0.99143 |

**Table S2.** Mean, standard deviation (SD),coefficient of variation (CV) and limit of detection (LOD).

| | | | | | | | |
|---|---|---|---|---|---|---|---|
| **Mean, SD CV, and LOD of sigmoidal fit curve analysis (Figure 4C,PBS)** | | | | | | | |
| **Sample (CFU/ml)** | 0 | $1.5x10^4$ | $1.5x10^5$ | $4.5x10^5$ | $9.0x10^5$ | $1.5x10^6$ | $1.5x10^7$ |
| **Mean (intensity)** | 6,886 | 10,402 | 25,476 | 40,510 | 55,900 | 71,251 | 71,613 |
| **SD** | 2,677 | 4,296 | 7,689 | 14,652 | 9,151 | 6,478 | 11,726 |
| **CV (%)** | 39 | 41 | 30 | 36 | 16 | 9 | 16 |
| **LOD (RFU)** | $y_{LOD}$ = (6,886 + 1.645 * 2,677) + 1.645 * 4,296 = 18,356 RFU | | | | | | |
| **LOD (CFU/ml)** | $1x10^5$ CFU/ml (95% confidence interval: $3.7x10^4$ – $2.5x10^5$ CFU/ml) | | | | | | |
| **Mean, SD, CV, and LOD of sigmoidal fit curve analysis (Figure 4C,wound exudate)** | | | | | | | |
| **Sample (CFU/ml)** | 0 | $1.5x10^4$ | $1.5x10^5$ | $4.5x10^5$ | $9.0x10^5$ | $1.5x10^6$ | $1.5x10^7$ |
| **Mean (intensity)** | 5,112 | 4,477 | 9,764 | 13,955 | 27,818 | 49,839 | 72,157 |
| **SD** | 1,368 | 1,599 | 1,983 | 4,350 | 10,818 | 8,649 | 8,120 |

| | | | | | | | |
|---|---|---|---|---|---|---|---|
| **CV (%)** | 27 | 36 | 20 | 31 | 39 | 17 | 11 |
| **LOD (RFU)** | $y_{LOD}$ = (5,112 + 1.645 * 1,368) + 1.645 * 1,599 = 9,993 RFU | | | | | | |
| **LOD (CFU/ml)** | 2.1x$10^5$ CFU/ml (95% confidence interval: 9.6x$10^4$ – 4.7x$10^5$ CFU/ml) | | | | | | |
| **Mean and standard deviation of linear regression line analysis (Figure S1)** | | | | | | | |
| **Time point (h)** | 3.5 | 4.0 | 4.5 | 5.0 | 5.5 | 6.0 | 6.5 |
| **Mean (CFU/ml)** | 7.9x$10^7$ | 1.3x$10^8$ | 1.7x$10^8$ | 2.4x$10^8$ | 3.6x$10^8$ | 4.7x$10^8$ | 5.5x$10^8$ |
| **SD (CFU/ml)** | 2.3x$10^7$ | 0.6x$10^8$ | 0.2x$10^8$ | 0.1x$10^8$ | 0.1x$10^8$ | 1.6x$10^8$ | 0.5x$10^8$ |
| **Mean (OD600)** | 0.19 | 0.27 | 0.34 | 0.43 | 0.53 | 0.63 | 0.74 |
| **SD (OD600)** | 0.00 | 0.00 | 0.00 | 0.01 | 0.01 | 0.00 | 0.02 |

| **Mean, SD, CV, and LOD of sigmoidal fit curve analysis (Figure S7A)** | | | | | | | | |
|---|---|---|---|---|---|---|---|---|
| **Sample (cp/reaction)** | NTC | 1x$10^1$ | 5x$10^1$ | 1x$10^2$ | 1x$10^3$ | 1x$10^4$ | 1x$10^5$ | 1x$10^6$ |
| **Mean (intensity)** | 6,420 | 4,466 | 8,462 | 12,617 | 42,884 | 79,977 | 72,393 | 75,062 |
| **SD** | 1,462 | 2,317 | 1,668 | 3,454 | 9,178 | 6,442 | 11,489 | 7,658 |
| **CV (%)** | 23 | 52 | 20 | 27 | 21 | 8 | 16 | 10 |
| **LOD (RFU)** | $y_{LOD}$ = (6,420 + 1.645 * 1,462) + 1.645 * 2,317 = 12,637 RFU | | | | | | | |
| **LOD (cp/reaction)** | 121 cp/reaction (95% confidence interval: 68 – 286 cp/reaction) | | | | | | | |
